# Circadian variation in plasma 5-fluorouracil concentrations during a 24 hour constant-rate infusion

**DOI:** 10.1186/s12885-015-1075-6

**Published:** 2015-02-18

**Authors:** Gini F Fleming, Philip Schumm, Greg Friberg, Mark J Ratain, Uchenna O Njiaju, Richard L Schilsky

**Affiliations:** 1 Section of Hematology/Oncology, Department of Medicine, 5841 South Maryland Avenue, MC 2115, Chicago, IL 60637 USA; 2 The University of Chicago Comprehensive Cancer Center, 5841 South Maryland Avenue, Chicago, IL 60637 USA; 3 Committee on Clinical Pharmacology and Pharmacogenomics, 57th Street Box 11, Chicago, IL 60637 USA; 4 Department of Public Health Sciences, The University of Chicago, 5841 S. Maryland Avenue, Chicago, IL 60637 USA; 5 The University of Chicago Medical Center, 5841 S. Maryland Ave., MC 2115, Chicago, IL 60637 USA

**Keywords:** Circadian, 5-fluorouracil, Cancer

## Abstract

**Background:**

Varying the rate of continuous intravenous infusions of 5-fluorouracil (5FU) chemotherapy over a 24-hour period has been reported to improve patient outcomes. It has been hypothesized that circadian variation in drug disposition is a contributing factor. We analyzed 5-FU concentrations during a 24-hour continuous 5-FU infusion.

**Methods:**

Sixty-four subjects with advanced malignancies including pancreatic, hepatocellular, colorectal as well as other epithelial malignancies and either abnormal hepatic or renal function were treated on a phase I and pharmacokinetic study of weekly 24-hour intravenous infusions of 5-FU and leucovorin. No other concomitant anticancer therapy was administered. Blood samples were collected every three hours from 61 subjects for measurement of plasma 5-FU during the first two weekly infusions.

**Results:**

After adjusting for differences in dose, elapsed time from start of infusion and infusion number (2 versus 1), mean 5-FU concentration was highest at 6 am and lowest at 3 pm, with an overall change in the mean from 3 pm to 6 am of +20 percent (95% CI = 12–28%). However, this variation in mean concentration associated with time of day was comparable in magnitude to the between-patient differences, within-patient differences between infusions, and the residual variation within infusion (coefficient of variation = 21%).

**Conclusions:**

Our data show systematic variation by time of day in plasma concentrations of 5-FU administered at a constant rate over 24 hours, but it is small compared to the total variation in plasma concentration contributed by other sources. Circadian variation in men was more pronounced than in women.

## Background

5-fluorouracil (5-FU) is a fluorinated pyrimidine with activity against several common solid malignancies. It is one of the oldest anticancer agents, but remains a mainstay of treatment, particularly for colorectal cancer. It is commonly given as a 48 hour continuous intravenous infusion in regimens such as FOLFOX (folinic acid/5-FU/oxaliplatin), FOLFIRI (folinic acid/5-FU/irinotecan), and FOLFIRINOX (folinic acid/5-FU/irinotecan/oxaliplatin). A randomized clinical trial showed that when 5-FU was given as a continuous intravenous infusion, varying the dose administered such that the maximal dose was administered around 04:00 a.m. (referred to as “chronomodulated therapy”) patients experienced a 5-fold reduction in the risk of severe mucosal toxicity compared to a constant rate infusion at the same dose [1]. Other reports have suggested that such chronomodulated therapy also enabled patients to receive higher doses of 5-FU than conventional continuous infusion regimens, with an improved toxicity profile [2]. As 5-FU has a short half-life and toxicity is known to be related to plasma concentration [3], one possible explanation for benefit from chronomodulated therapy is that plasma levels of 5-FU vary systematically with time of day despite a constant dose-rate infusion and either exaggerating or abrogating this variability might be beneficial. The variation in 5-FU levels has been hypothesized to be related to circadian variation in the activity of dihydropyrimidine dehydrogenase (DPD), the rate-limiting enzyme in 5–FU catabolism [4,5].

Several other studies have evaluated circadian variation in 5-FU levels during continuous intravenous infusion, and they have reported conflicting results (Table 1). Some found no circadian variability at all, and of those that did, most did not find similar times of day for peak and trough. All reports published to date included relatively small numbers of subjects and the sampling frequency varied. As part of a phase I study of a 24-hr 5-FU and leucovorin infusion in patients with organ dysfunction conducted during the 1990s, we performed pharmacokinetic sampling every three hours. We now analyze this dataset to examine potential variations in 5-FU levels by time of day. This represents by far the largest dataset examining potential circadian fluctuation in 5-FU plasma levels with a constant-rate infusion of drug, and is the first to include a substantial number of patients with lower performance status (ECOG 2) and organ impairment, including assessing possible effects of organ impairment on diurnal changes in 5-FU levels. In addition, it is also the first to compare the magnitude of the circadian effect to other sources of variation in 5FU levels, such as between-subject, within-subject between-cycle and residual variability.

**Table 1:** **Prior studies of circadian variability in 5-fluorouracil plasma levels during CVI**

Reference	Pts	5-FU dose (mg/m^2^/d)	Sampling (hours)	Data analysis	Peak time	Trough time	Estimated [5-FU] change (peak to trough)
Petit [6]	7	450-966	Q3	ANOVA of mean transformed [5-FU] by time (% change from pt mean)	1:00	13:00	−43% (2× amplitude from cosinor analysis)
Harris [4]	7	300	Q3	Cosinor analysis of transformed [5-FU] (% change from pt mean)	11:00	23:00	−80% (mean of individual patient peaks and troughs)
Sparano [7]	15	150-300	Q1-16	Cosinor analysis of transformed [5-FU] (% change from pt mean)	8:00	20:00	−2.5 standard deviations from individual’s mean [5-FU] (at individual’s peak and trough)
Metzger [8]	4	600	Q6	ANOVA of mean [5-FU] by time	4:00	13:00	−88% (mean [5-FU] at the population’s peak and trough)
Van Kuilenburg [9]	5	300-450	Q4	Not stated	No circadian variation observed		
Strocchi [10]	9	600	Q3	Not stated	3 of 9 pts had “marked circadian variation” but peak was variable		
Takimoto [11]	14	1758	Q3	ANOVA of mean [5-FU] by time (Kruskal-Wallis test)	No consistent circadian variation observed; 5 Fu concentrations within individuals varied 1.7 fold		
Kuwahara [12]	35	400-550	Q12	Comparison of two timepoints	Higher concentrations observed at 17:00 and lower at 5:00. Difference observed only at lower dosage.		

## Methods

### Study subjects

Eligibility criteria, patient characteristics and diagnoses for patients on the phase I trial have been previously reported [13]; the most relevant characteristics for those included in this analysis of plasma 5-FU concentrations are shown in Table 2. The most common tumor types were pancreatic, hepatocellular and colorectal cancer, but a large number of other epithelial malignancies were included. According to the design of the phase I trial, patients were prospectively classified according to degree of organ dysfunction: Cohort I, creatinine >1.5 mg/dL but ≤3.0 mg/dL, and normal bilirubin (referred to as the high creatinine group); Cohort II, bilirubin >1.5 mg/dL but <5.0 mg/dL, with normal creatinine (intermediate bilirubin group); or Cohort III, bilirubin ≥5.0 mg/dL with normal creatinine (high bilirubin group). Cohort IV consisted of five patients whose organ function normalized during the period between registration and treatment initiation. 5-FU doses were escalated separately in groups of at least three patients in each organ dysfunction cohort, from 1000 mg/m^2^ to 1800 mg/m^2^ to 2600 mg/m^2^. There was no intra-individual dose escalation. Data on 5-FU concentrations were not available for two patients and a pump malfunction occurred in a third, so our analysis includes only 61 of the 64 patients registered on the study. The University of Chicago Institutional Review Board approved the protocol and written informed consent was obtained from all patients enrolled in the study.

**Table 2:** **Patient characteristics, separately by organ dysfunction cohort (n = 61)**

*Characteristic*	Hi creat	Int bili	Hi bili	Normalized
No. of patients	16	15	25	5
Age, years, median (range)	66(44–76)	46(22–69)	56(22–79)	56(48–67)
Sex (no. of patients)				
Male	11	7	14	4
Female	5	8	11	1
Performance status (WHO)				
0	4	2	3	1
1	6	7	7	2
2	6	6	15	2

### Treatment

5-FU was mixed together with 500 mg/m^2^ leucovorin in 1000 ml 5% dextrose in water and administered weekly as a 24-hr continuous intravenous infusion. The first two infusions were administered in the General Clinical Research Center at the University of Chicago. Patients were randomly assigned to begin the first infusion at either 6 am or 6 pm, with the second infusion beginning at the alternate time point; this allowed us to distinguish between differences in 5-FU concentration due to time of day versus changes in concentration across the duration of the infusion. Blood for the measurement of 5-FU plasma concentrations was obtained prior to the start of treatment and then every 3 hr for the first and second weekly infusions. Blood for WBC measurement was sampled after admission to the research unit but prior to initiation of 5-FU infusion.

### Dose levels

The number of patients treated at each dose level has been previously reported in detail [13]. A total of 17, 21, and 26 patients were treated at dose levels of 1000 mg/m^2^, 1800 mg/m^2^, and 2600 mg/m^2^, respectively, the latter being the recommended 5-FU dose for this regimen in patients with normal hepatic and renal function [14]. Since 5-FU is not predominantly metabolized in liver or excreted by the kidneys (the highest tissue expression of DPD is in leucocytes [15]), we did not expect to find differences in 5-FU clearance between the organ dysfunction groups, and none were observed.

### 5-FU plasma concentrations

Seven to ten ml of blood was drawn into a heparinized tube every 3 hr during the first two 24-hr infusions. According to the protocol, the final sample was to be taken at the end of the infusion, and since several of these already reflected a marked drop in concentration, all final samples were excluded from analysis. Samples were centrifuged promptly, and the plasma was removed and stored at −80°C until analysis by high performance liquid chromatography assay. A modification of a standard assay was used [16]. In brief, proteins (plasma 1 mL) were precipitated with trichloroacetic acid 100 μL and the supernatant was extracted with 8 mL of ethyl acetate. The sample was then dried with nitrogen and reconstituted with 220 μL of 0.1 N sodium hydroxide. Samples (30 μL) were injected on two Beckman Ultrasphere ODS columns (inside diameter 4.6 mL and length 25 cm) which were connected in series. The mobile phase was sodium perchlorate 3 mM, pH 3, at 1.2 mL/min. The internal standard was 100 μL of 0.1 M bromouracil with a detection wavelength of 254 nm. The lower limit of quantification was 49.4 ng/mL. Intra-assay reproducibility (CV = 0.4-4.7%) and accuracy (range: 97.4-102.3) were determined by performing three measurements of the same six standards on the same day. Inter-assay reproducibility (%CV = 1.3-5.1) and accuracy (range: 96.7-102.3) were determined by assays of the same six standards in triplicate on two days.

### Data analysis

A Generalized Linear Mixed Model (GLMM) [17] was fit to the 5-FU concentrations for the first two infusions, using a log link and gamma variance function. This model specifies that the residual standard deviation around each patient-specific profile for a given infusion is proportional to the mean value (i.e., the coefficient of variation is constant). Covariates included dose, infusion (2nd vs.1st), elapsed time (in hours) from the start of the infusion, and an interaction between dose and elapsed time. Circadian variation was captured by including time of day, parameterized via a series of 8 contrasts capturing deviations from the overall linear trend throughout the infusion (i.e., parameters sum to zero). The model also includes a random patient effect (corresponding to differences in overall level between patients) and a random effect capturing within patient differences between infusions; these random effects were assumed to be normally distributed and uncorrelated. Estimated changes in the linear predictor were exponentiated to obtain the corresponding percentage change in the mean. Finally, cohort and gender were each added to the model one at a time, together with an interaction between them and time of day in order to determine whether the amount of circadian variation differed by group. The model was fit using the meglm command in Stata 13 [18].

In addition, a second model was fit to the data in which the circadian variation was modeled using a cosine curve with 24 hour period, allowing for a different amplitude and acrophase for each subject (a second harmonic with period 12 hours was also considered, but was found not to be statistically significant). All other components of the linear predictor (including both fixed and random effects) were identical to those in the first model. After using the linearizing transformation described in Mikulich et al. [19], the resulting linear mixed model was fit to the natural log of the concentrations (the log transformation was used to stabilize the variance). Best Linear Unbiased Predictions (BLUPs) of the random effects corresponding to the circadian component of the model were then back-transformed to yield subject-specific estimates of amplitude and acrophase. These were then regressed on gender, age, organ dysfunction cohort and performance status.

## Results

### Plasma 5-FU concentration

Results from the model fit to 5-FU concentrations for the first two infusions among 61 patients are presented in Table 3. At the higher two doses, mean concentration increased steadily throughout the infusion (likelihood ratio statistic for testing the interaction between dose and elapsed time was 24.0 on 2 d.f., p < 0.001); over the full 24 hours, it increased by 35% (95% CI = 21–52%) in the 1800 mg/m^2^ group and 54% (95% CI = 40–69%) in the 2600 mg/m^2^ group. In addition, concentrations during the second infusion were on average 8% higher (p = 0.058, 95% CI = 0–16%) than those during the first infusion.

**Table 3:** **Estimates from generalized linear mixed model** ^**†**^ **fit to 5FU concentrations (ng/ml)**

*Parameter*	Estimate	95% CI
Dose (mg/m^2^)		
1800 vs. 1000	0.292	(0.076, 0.508)
2600 vs. 1000	0.628	(0.429, 0.827)
Elapsed time (hours)		
1000 mg/m^2^	0.002	(−0.003, 0.007)
1800 mg/m^2^	0.013	(0.008, 0.017)
2600 mg/m^2^	0.018	(0.014, 0.022)
Infusion (2 vs. 1)	0.072	(−0.002, 0.147)
Time of day^§^		
00:00	0.033	(−0.005, 0.071)
03:00	0.058	(0.019, 0.096)
06:00	0.091	(0.039, 0.142)
09:00	−0.000	(−0.039, 0.038)
12:00	−0.018	(−0.056, 0.020)
15:00	−0.089	(−0.128, −0.049)
18:00	−0.040	(−0.094, 0.014)
21:00	−0.034	(−0.073, 0.005)
Constant	5.958	(5.794, 6.122)
SD of patient-level effects	0.264	(0.207, 0.338)
SD of within-patient, infusion effects	0.174	(0.136, 0.223)
Coefficient of variation	0.210	(0.198, 0.221)

^†^Generalized linear mixed model with log link and gamma variance function (i.e., constant coefficient of variation), fit to q3hr 5FU concentrations during first two 24 hr infusions. Model includes random effects for patient and infusion within patient.

^§^Parameters sum to zero, thus reflecting deviations from the linear trend over time within a single infusion.

**Figure 1:**
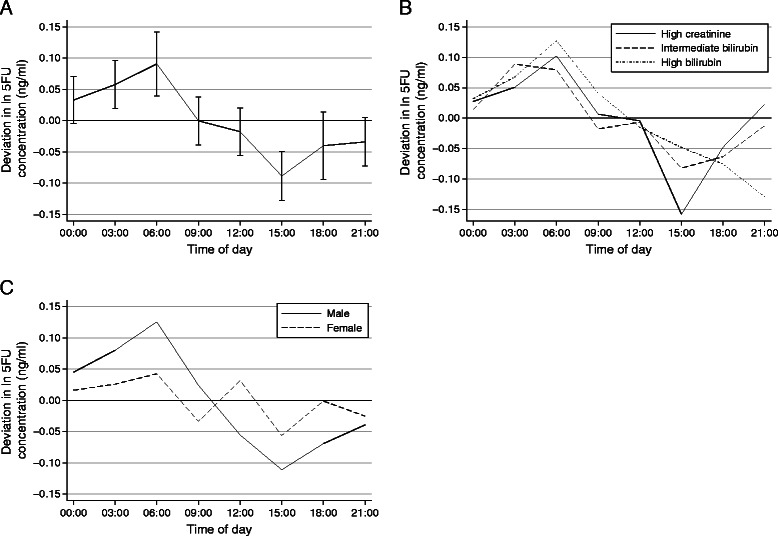
**Estimated changes in mean 5FU concentration (ng/ml) with time of day, obtained by fitting a generalized linear mixed model (GLMM) to the data from 61 patients (two 24-hr infusions each).** Panel **A** shows estimates for the overall sample, together with 95% confidence intervals. Panel **B** shows estimates for three cohorts, from a model adding cohort and an interaction between cohort and time of day. Panel **C** shows estimates for men and women, from a model adding sex and an interaction between sex and time of day.

Controlling for elapsed time, a circadian pattern in mean 5-FU concentration was observed (likelihood ratio statistic 42.5 on 7 d.f., p < 0.001). The estimated mean deviations from the underlying linear trend for each three hour interval are shown in Table 3 and plotted in Figure 1A. The lowest mean concentration occurred at 3 pm and the highest at 6 am, representing an overall increase in concentration from lowest to highest of 20% (95% CI = 12–28%). This same pattern was evident among both the infusions that began at 6 am and those that began at 6 pm. A similar circadian pattern was observed in all three organ dysfunction cohorts (Figure 1B), with the minor differences in the observed profiles consistent with random variability (likelihood ratio statistic for testing interaction 18.6 on 14 d.f., p = 0.18). In contrast, men exhibited greater circadian variation than women (Figure 1C; likelihood ratio statistic 13.7 on 7 d.f., p = 0.058), with an increase in 5-FU concentration from 3 pm to 6 am of 27% (95% CI = 16–39%) as compared to only 10% (95% CI = −1–23%) for women.

After accounting for the systematic differences in 5-FU concentration associated with dose, elapsed time, infusion number and time of day, considerable variability both between and within patients still remained. The largest source of variation was between patients, with an estimated standard deviation on the log scale of 0.26 for the patient effect (95% CI = 0.21–0.34), corresponding to an increase in 5-FU concentration of 30 percent (these differences are in addition to the systematic differences between dose levels, which are already included in the model). A smaller but still significant amount of variability was observed within patients between the two infusions; the standard deviation on the log scale of these infusion-level effects was 0.17 (95% CI = 0.14–0.22), corresponding to an increase in 5-FU concentration of 19 percent. Finally, the residual variability (within patient and infusion) had an estimated coefficient of variation of 21%, indicating that there is as much variability remaining even after all other sources have been accounted for as there is due to the circadian pattern.

**Figure 2:**
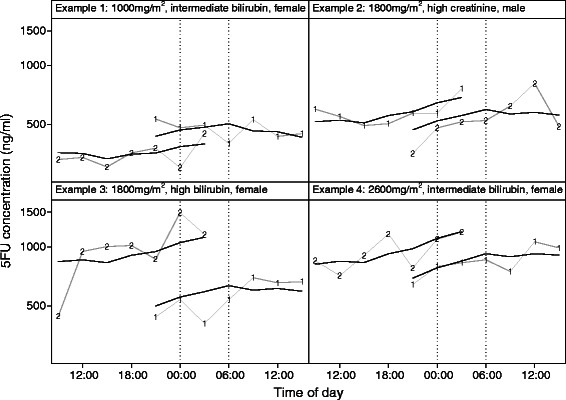
**Observed 5FU concentrations (in ng/ml, labeled with cycle number and connected by gray lines) together with fitted values from a generalized linear mixed model (solid black lines) for four selected patients (two cycles each).** Fitted values include empirical Bayes (posterior means) estimates of random effects for patient and for cycle within patient. Dashed lines indicate the period between midnight and 6 a.m. when highest *average* 5-FU concentrations were observed.

The magnitude of the random variation within patients between infusions and of the residual variation relative to the systematic effects (i.e., the increase with elapsed time and the circadian pattern) captured by the model is illustrated in Figure 2, which plots the observed 5-FU concentrations (connected by gray lines) and fitted values (including the posterior means of the random effects) from the model for four actual patients. The daily period with highest *average* 5-FU concentrations (midnight through 6 am) is indicated by the dotted vertical lines. Note that in only 4 of the 8 cycles shown did the highest observed concentration occur between midnight and 6 am, while among the other 4 cycles, some of the lowest concentrations occurred during this period.

Results based on the cosine model fit to the log concentrations were very similar to those in Table 3. Specifically, estimates of the effects of dose, elapsed time and their interaction were nearly identical, as were the estimated between-subject and within-subject between-cycle variance components. The residual variability was slightly smaller as some of it was accounted for by the subject-specific cosine curves, however the predictions from this model and the first model had a correlation of 0.99, reflecting the close correspondence between the two. The mean amplitude for the entire sample was 0.10, which corresponds to an increase in 5FU concentration from trough to peak of 22%. The standard deviation of the amplitudes was 0.06, which translates into increases in concentration from trough to peak of just 8% for subjects with amplitudes one standard deviation below the mean, and 38% for subjects with amplitudes one standard deviation above the mean. Similarly, the standard deviation of the acrophases was 1.09, corresponding to a time shift of approximately 4.2 hours. Consistent with the results above, the mean amplitude was higher for men than for women, and this difference was statistically significant despite adjusting for age, organ dysfunction cohort and performance status (p = 0.004). However, there were no other statistically significant relationships between these covariates and either the amplitude or acrophase. In particular, the standard deviation of the acrophases was nearly identical for men and women (1.12 versus 1.08), providing further evidence that the gender difference in Figure 1C reflects a true difference in amplitude rather than simply greater variability in the acrophase among women.

## Discussion

We observed a circadian pattern in mean 5-FU concentrations over a 24-hr continuous infusion, with the highest concentrations observed in the early morning and the lowest concentrations in the afternoon. We did not find any variation in magnitude of circadian variation by renal or liver dysfunction category. It has previously been reported that patients with cirrhosis had a delay in peak cortisol times by about 1.5 hours [20], however the resolution of our sampling (every three hours) would not permit detection of such a difference. Disturbances in chronoregulated variables such as diurnal melatonin secretion have been reported in end-stage renal disease [21]; these are most pronounced in patients with end-stage renal disease on dialysis, and we did not anticipate changes in our subjects who all had creatinine ≤ 3.0 mg/dL.

The circadian pattern in 5-FU levels was more pronounced in men than in women, consistent with published data that both 5-FU metabolism (women have slower 5-FU elimination) [22] and circadian timing systems [23] differ by gender. Interestingly, a meta-analysis of three phase III trials in patients with metastatic colorectal cancer found an overall survival benefit to the chronomodulated schedule for men, but not for women [24]. However, as we show here, systematic variation by time of day accounts for only a small part of the total variation in 5-FU concentrations during a 24-hr infusion, and individual maximum and minimum 5-FU concentrations for a given infusion are not generally predictable or reproducible across cycles for a given patient.

It is possible that circadian variation in 5-FU concentration is due, at least in part, directly to factors associated with time-of-day such as activity, food, light, or sleep, rather than—or interacting with—an intrinsic periodicity of metabolism [25]. This could account for the variability in results seen across series assessing the relationship of 5-FU concentrations with time of day, and would also increase the likelihood of detecting circadian variation in our series, where all patients were housed in a clinical research unit where meal timing was fairly uniform and efforts were made to maintain quiet at night. Variability across the studies in Table 1 might also be related to the fact that in some of the studies the 5-FU infusions were given along with other chemotherapy drugs, such as cisplatin [6] or PALA (N-(phosphonacetyl)-L-aspartate) [11] that could have had an impact on 5-FU clearance. It has been reported, for example, that irinotecan alters the circadian rhythm of dihydropyrimidine dehydrogenase mRNA in mouse liver [26]. Time from start of infusion might also have confounded some series; by randomly assigning patients to begin their first cycle at either 6 a.m. or 6 p.m. and then alternating for the second infusion, we were able to adjust for the steady rise in 5-FU levels that was observed with the higher dose infusions. All studies were consistent in observing substantial random fluctuation in 5-FU dose across the span of a continuous dose-rate infusion, and this needs to be taken into account in study designs that dose-adjust 5-FU levels of continuous infusions based on a plasma concentration [3].

Plasma 5-FU concentrations increased steadily over the 24-hr infusion at the two highest doses (evident in Examples 2–4 in Figure 2). This is likely due to the well-known non-linear elimination kinetics of 5-FU, which are thought to be related to saturation of metabolic or transport processes. We also found that mean 5-FU concentrations were higher during the second infusion a week later for the same patient, although only by 8%; higher 5-FU concentrations during a subsequent cycle were also observed by Kuwahara et al [12]. White blood cells, which have the highest level of DPD expression, are known to have a diurnal variation in number [27]. We compared WBC numbers for cycles 1 and 2 to see if a difference in WBC might account for the difference in 5FU concentrations between cycles, but there was no difference in WBC number between cycles #1 and 2 (data not shown).

Given the fact that systemic time-of-day variability in plasma 5-FU levels accounts for a relatively small percentage of the overall variability in 5-FU levels within each individual during a continuous rate infusion, we would suggest that the benefit from chronomodulation of 5-FU observed in clinical trials is unlikely to be primarily related to time-of-day differences in total-body 5-FU clearance. Other factors that can account for differences in drug toxicity or efficacy based on time of day of administration include circadian variation in normal tissue proliferation or tissue-specific metabolism. For example, it has been shown that there is significant circadian variation in both DPD and thymidylate synthase mRNA expression in rat jejunum, with a peak at early rest phase corresponding roughly to time of lower GI adverse effects from 5-FU in human trials (sleep) [28]. Cell proliferation in human bone marrow and gut has likewise been shown to exhibit profound circadian variability [29,30]. Circadian timing of chemotherapy may exploit differences in proliferation and/or drug metabolic rhythms between normal and malignant tissues.

## Conclusions

We conclude that circadian variation in 5-FU metabolism is real, and persists in patients with advanced stage metastatic cancer, even those with hepatic and renal dysfunction. Moreover, it is more pronounced in men than in women. However, this variation represents only a small part of the overall variation in 5-FU levels during a constant dose-rate infusion.

## Abbreviations

5-FU: 5Fluorouracil
Mg: Milligrams
dL: Deciliters
m^2^: Meters squared
hr: Hour
mL: Milliliters
mM: Millimolar
N: Normal
CV: Coefficient of variation
ng: Nanograms
vs: Versus
d.f.: Degrees of freedom
CI: Confidence interval
DPD: Dihydropyrimidine dehydrogenase
GI: Gastrointestinal
Creat: Creatinine
Bili: Bilirubin
CVI: Continuous venous infusion
